# Comment on “Revisiting the Relationship Between Legal Awareness and Safe Abortion Utilization”

**DOI:** 10.1002/hsr2.73407

**Published:** 2026-10-22

**Authors:** Muhammad Ahmad khan, Nida sher

**Affiliations:** ^1^ Nowshera Medical College Nowshera Pakistan; ^2^ Khyber Girls Medical College Peshawar Pakistan

We read with interest the recent article by Banchani et al. On the link between knowledge of the legality of abortion and utilization of safe abortion for women in Ghana [1]. The authors are commended for their efforts in tackling an important public health issue and for their use of nationally representative data to examine the potential role of legal awareness and educational attainment in promoting safe abortion practices. The results offer important evidence of the need for reproductive health literacy. We nonetheless want to draw attention to two methodological aspects that could indicate a cautious interpretation of the reported associations.

First, it is unclear whether the timing of awareness of the legalization of abortions is correlated with subsequent uptake of safe abortions. While the authors considered legal awareness to be a factor associated with safer abortion practices, both the exposure (awareness of the legality of abortion) and outcome were measured at the time of survey and not at the time of the abortion. Accordingly, it is difficult to determine if women were already familiar with the laws before deciding on abortion care. Women who had abortions at formal health service providers could have been given counseling or information on abortion law during their interactions with the health service providers, and thus have learned the law after having an abortion. Women who had abortions at formal health service providers could have been advised or given information on abortion law during their interactions with the health service providers and have thus gained legal awareness after abortion. This option raises the issue of reverse causation and makes it difficult to conclude whether there was any association between legal awareness and abortion care‐seeking behavior [2, 3].

Second, the study was based on self‐report of abortions during the past 5 years. The longer the recall period, the higher the chances of recall bias, especially for information concerning abortion methods, providers, health care facilities, and prior knowledge of the legality of abortion procedures. Additionally, awareness of abortion law can change over time through the process of education, media exposure or encountering health services. So the legal awareness that is reported in the survey may not necessarily provide a true picture of the respondent's level of awareness at the time of the abortion decision. This difference may have led to misclassification of exposure and affected the estimated exposure‐safe abortion association [4, 5].

To sum up, the study offers country‐wide representative data on the association between the knowledge of the legal access to abortion and uptake of safe abortion in Ghana. We comment on the results with the hope that readers will be aware of the possibility of temporal ambiguity between exposure and outcome measurements, and of recall bias in self‐reported exposures. Conducting future prospective or longitudinal studies that examine legal awareness prior to decisions about abortion would help clarify the direction of the association observed in this study and would provide greater evidence of the impact of legal awareness on access to safe abortion services.

## Author Contributions

**Muhammad Ahmad Khan:** conceptualization, methodology, data curation, investigation, formal analysis. **Nida Sher:** validation, supervision, visualization, writing – original draft, writing – review and editing.

## Funding

The authors have nothing to report.

## Ethics Statement

The authors have nothing to report.

## Consent

The authors have nothing to report.

## Conflicts of Interest

The authors declare no conflicts of interest.

## Data Availability

No new data were analyzed or generated in support of this research.
